# Murine Models and Human Cell Line Models to Study Altered Dynamics of Ovarian Follicles in Polycystic Ovary Syndrome

**DOI:** 10.1002/adbi.202400713

**Published:** 2025-01-22

**Authors:** Arturo Bevilacqua, Cristiano Giuliani, Giovanna Di Emidio, Samuel H Myers, Vittorio Unfer, Carla Tatone

**Affiliations:** ^1^ Department of Dynamic and Clinical Psychology, and Health Studies Sapienza University of Rome Via Dei Marsi 78 Rome 00185 Italy; ^2^ The Experts Group on Inositols in Basic and Clinical Research and on PCOS (EGOI‐PCOS) Rome Italy; ^3^ Systems Biology Group Lab and Research Center in Neurobiology Daniel Bovet (CRiN) Rome 00185 Italy; ^4^ Department of Life Health and Environmental Sciences University of L'Aquila L'Aquila 67100 Italy; ^5^ R&D Department Lo.Li Pharma s.r.l Rome 00156 Italy; ^6^ UniCamillus‐Saint Camillus International University of Health Sciences Rome 00156 Italy

**Keywords:** animal models, cellular models, d‐chiro‐inositol, granulosa cells, hyperandrogenism, ovarian dysfunction, polycystic ovary syndrome (PCOS)

## Abstract

Polycystic ovary syndrome is one of the most common endocrine disorders in women of reproductive age, characterized by functional and structural alterations of the female reproductive organs. Due to the unknown underlying molecular mechanisms, in vivo murine models and in vitro human cellular models are developed to study the syndrome. These models are used to analyze various aspects of the pathology by replicating the conditions of the syndrome. Even though the complexity of polycystic ovary syndrome and the challenge of reproducing all its features leave several questions unanswered, studies conducted to date have elucidated some of the alterations in ovarian follicle molecular and cellular mechanisms involved in the syndrome, and do not require the employment of complex and invasive techniques on human patients. This review examines ovarian functions and their alterations in polycystic ovary syndrome, explores preclinical in vivo and in vitro models, and highlights emerging research and medical perspectives. It targets researchers, healthcare professionals, and academics, including endocrinologists, cell biologists, and reproductive medicine specialists, studying the molecular and cellular mechanisms of the syndrome.

## Graphical abstract

The manuscript examines preclinical murine and human models to study polycystic ovary syndrome (PCOS), delving into the cellular and molecular mechanisms underlying altered ovarian follicular dynamics. It explores the cellular interactions involved in normal and PCOS ovaries and outlines the current and novel strategies in the search for preclinical information and potential therapeutic approaches. It thus offers fresh perspectives in endocrinological and reproductive research.

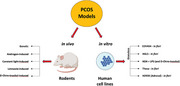

## 1 Introduction

Polycystic ovary syndrome (PCOS) is an endocrine disorder that affects ≈10–13% of women of reproductive age and is significantly associated with infertility.^[^ ^1^ ^]^ PCOS is characterized by chronic inflammation and oxidative stress, and its etiology, while still not fully understood, is believed to involve familial and genetic factors,^[^ ^2^ ^]^ hormonal and/or metabolic imbalances,^[^ ^3^ ^]^ in addition to obesity often coupled with insulin resistance.^[^ ^4^ ^]^

It was first described by Stein and Leventhal in 1935 among women with hirsutism, obesity, amenorrhea, and bilateral enlarged polycystic ovaries.^[^ ^5^ ^]^ With time, several diagnostic criteria have been proposed. These criteria include combinations of amenorrhea or oligomenorrhea, hyperandrogenism, and changes in ovarian morphology as assessed by pelvic ultrasonography. In 2003, the American Society for Reproductive Medicine (ASRM) and the European Society for Human Reproduction and Embryology (ESHRE) established the Rotterdam criteria for diagnosing PCOS, requiring two of the following three key characteristics: oligo/anovulation, clinical and/or biochemical hyperandrogenism, and polycystic ovarian morphology.^[^ ^6^ ^]^ In 2006, the Androgen Excess and PCOS Society (AE‐PCOS) highlighted hyperandrogenism as a fundamental hallmark of PCOS.^[^ ^7^ ^]^

Although the term PCOS suggests that the presence of ovarian cysts is essential for diagnosis, other symptoms of PCOS can persist even after complete removal of the ovaries, and not all women with polycystic ovaries exhibit the syndrome.^[^ ^8^ ^]^ Consequently, many clinicians who previously viewed PCOS as a gynecological issue now recognize that it is a multisystem disorder primarily involving hormonal and metabolic dysregulation, insulin resistance, and hyperandrogenism.^[^ ^9^ ^]^ For this reason, several authors have proposed a reassessment of the diagnostic criteria of the syndrome.^[^ ^10^ ^]^

Hyperandrogenism arises from an imbalance between androgen production in ovarian theca cells, and their subsequent conversion into estrogens, which occurs in granulosa cells.^[^ ^11^ ^]^ Elevated androgen levels typical of PCOS trigger inflammatory processes with the expression of inflammatory cytokines in the ovaries, which can cause mitochondrial damage leading to follicle cell apoptosis, follicular dysplasia, and ovulation failure.^[^ ^12^, ^13^ ^]^ One primary cause of hyperandrogenism in PCOS is insulin resistance, which leads to elevated insulin levels that stimulate ovarian androgen secretion and suppress hepatic production of sex hormone‐binding globulin (SHBG).^[^ ^14^ ^]^ Increased androgen production by theca cells contributes to the clinical manifestations of PCOS, including impaired follicular development and excessive levels of circulating androgens.^[^ ^15^ ^]^ Understanding the roles of theca cells and granulosa cells in both healthy and PCOS‐affected ovaries is crucial for advancing PCOS research.

## 2 The Theca Cell‐Granulosa Cell Interplay in Steroidogenesis

Theca cells and granulosa cells are the somatic components of the ovarian follicle and are involved in steroidogenesis. They interact in a coordinated manner under the influence of various hormones and growth factors to produce the steroid hormones that are necessary for follicular development and ovulation. Theca cells originate from cortical stromal cells in the ovary and differentiate into either steroidogenic cells or fibroblasts/perivascular cells in response to paracrine signals from oocytes and granulosa cells/cumulus cells. Within an activated follicle during the ovarian cycle, these cells begin to proliferate and differentiate into an inner layer, the *theca interna*, and an outer layer, the *theca externa*. Theca interna cells include vascularized steroidogenic cells located near the basal lamina,^[^ ^16^ ^]^ while theca externa cells consist of non‐steroidogenic cells in contact with the ovarian stroma.^[^ ^17^ ^]^ Once differentiated, steroidogenic theca interna cells become responsive to luteinizing hormone (LH) as well as other endocrine and intra‐ovarian signals, synthesizing androgens that diffuse into granulosa cells and become substrates for estrogen synthesis.^[^ ^18^ ^]^ Theca cells also secrete factors with autocrine effects, and paracrine effects on granulosa cells.

**Figure 1:**
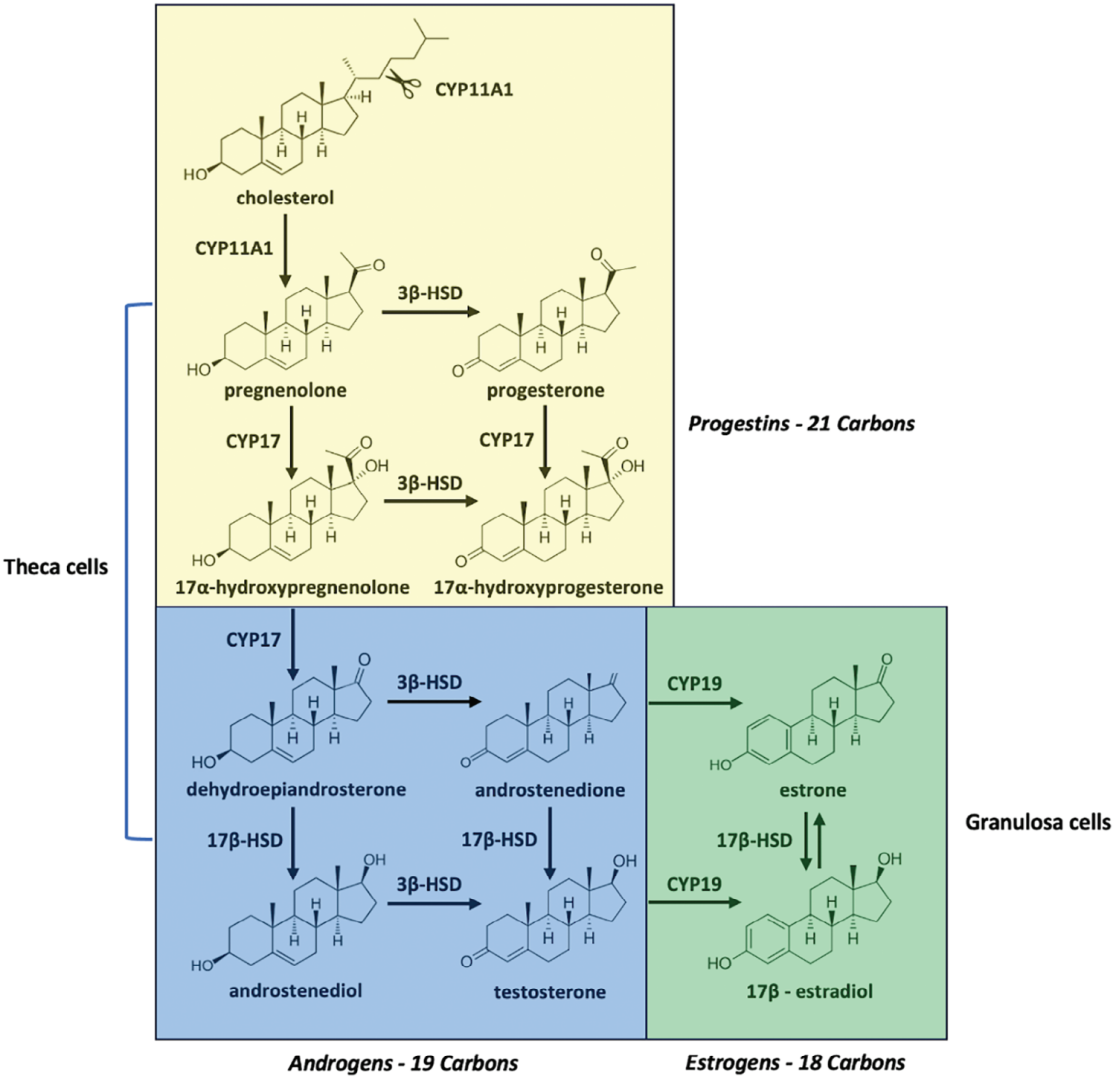
Map of Ovarian Steroidogenesis, with metabolic pathways and enzymes involved in the biosynthesis of steroids in theca and granulosa follicle cells: CYP11A (Cholesterol side‐chain cleavage enzyme; Cholesterol Monooxygenase; Steroid 20‐22‐Desmolase; Steroid 20‐22‐Lyase), CYP17 (17α‐hydroxylase/17,20‐lyase), 3β‐HSD (3β‐hydroxysteroid‐dehydrogenases), 17β‐HSD (17β‐hydroxysteroid‐dehydrogenases), CYP19 (Aromatase). Steroid categories are based on the numbers of Carbon atoms, chemical structures, and biological functions. Data from Conley and Bird (1997); Duranova et al. (2022).^[^ ^26^, ^27^ ^]^

LH is released from gonadotropic cells of anterior pituitary in response to hypothalamic surges of gonadotropin‐releasing hormone (GnRH).^[^ ^19^ ^]^ Under LH stimulation, theca cells synthesize androgens via induction of the expression of the *Steroidogenic Acute Regulatory Protein (StAR)* gene, *and Cytochrome P450 superfamily gene members 11A1 (CYP11A1)* and *17A1* (*CYP17A1)*. StAR and CYP11A1 facilitate the transport of cholesterol into the mitochondria and its conversion into pregnenolone, respectively. Pregnenolone is then transferred to the endoplasmic reticulum and converted to dehydroepiandrosterone (DHEA) and androstenediol by CYP17A1. Androstenedione, derived from DHEA, and androstenediol are then converted into testosterone by additional enzymatic reactions (**Figure** **1**).

**Figure 2:**
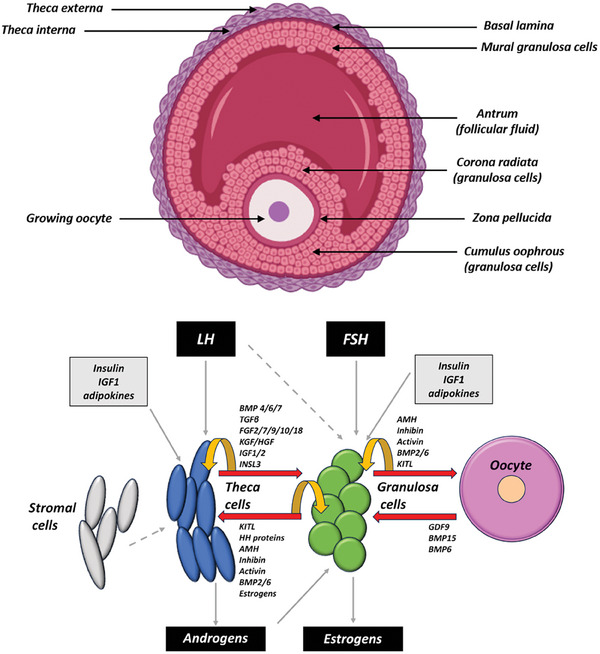
Top: Schematic representation of an antral ovarian follicle showing theca layers, basal lamina, granulosa cells, antrum, zona pellucida, *cumulus oophorus*, and growing oocyte. Bottom: Regulation of ovarian steroidogenesis. AMH (anti‐mullerian hormone), BMP (bone morphogenetic protein), IGF (insulin‐like growth factor), INSL3 (insulin‐like peptide 3), GDF (growth and differentiation factor), HGF (hepatocyte growth factor), HH proteins (hedgehog proteins), KITL (kit ligand or stem cell factor), and TGF (transforming growth factor). Theca cells, colored in blue, respond primarily to luteinizing hormone (LH). These cells produce androgens and are influenced by factors that include BMP4/6/7, TGFβ, FGF, KITL, and IGF1/2. Granulosa cells, colored in green, are regulated by follicle‐stimulating hormone (FSH) and convert theca cells androgens into estrogens. They secrete, and respond to, various molecules like AMH, inhibins, activins, BMP2/6, and c‐kit ligand (KITL), which facilitate oocyte maturation. Oocyte represented in pink communicates with granulosa cells by secreting factors like GDF9, BMP15, and BMP6, which are critical for follicular development. Arrows represent paracrine effects (red), autocrine effects (yellow), and endocrine effects (solid and dashed grey). Modified from Knight and Glister, 2019.^[^ ^18^ ^]^ (Composed in Biorender.com).

The frequency of LH release is regulated by steroid feedback loops, which vary throughout the ovarian cycle and are influenced by external factors such as the photoperiod and other environmental/social signals. LH binds to theca cell membrane receptors, triggering a signaling cascade that activates the expression of enzymes involved in steroidogenesis. It has been shown that inhibiting LH release via a GnRH antagonist reduces StAR and CYP17A1 mRNA levels in theca cells, leading to decreased androgen production.^[^ ^20^ ^]^ Ovarian folliculogenesis is also regulated by metabolic pathways; for example, insulin stimulates androgen production in theca cells,^[^ ^21^ ^]^ while adipokines such as leptin and adiponectin have an inhibitory role on androgen production, as observed in cultured bovine theca cells.^[^ ^22^ ^]^ Members of the transforming growth factor beta family (TGFβ) such as TGF‐β, bone morphogenetic proteins (BMPs), and activins, also modulate steroidogenesis at the theca cell level (**Figure** **2**).^[^ ^23^ ^]^

Once diffused into granulosa cells, theca cell androgens become the substrate for estrogen synthesis. This reaction is catalyzed by the enzyme aromatase, encoded by the *Cytochrome P450 superfamily gene member 19A1 (CYP19A1)*.^[^ ^24^ ^]^ Specifically, androstenedione is converted into estrone, while testosterone is converted into estradiol. These reactions are regulated by follicle‐stimulating hormone (FSH) signaling and provide granulosa cells with a central, crucial role in follicle growth and ovarian functions (Figure 2).^[^ ^25^ ^]^

Granulosa cells originate from somatic cells surrounding the oocyte in primordial ovarian follicles. They initially proliferate in a hormone‐independent manner but soon become stimulated by gonadotropins, especially FSH. Key factors in granulosa cell proliferation include follistatin, BMPs, the transcription factor forkhead box L2 (FOXL2),^[^ ^28^ ^]^ insulin‐like growth factor‐1 (IGF1), and activin,^[^ ^29^, ^30^ ^]^ which act by increasing the expression of cyclin D2, a key regulator of the cell cycle, and stimulating DNA synthesis.^[^ ^31^ ^]^

Under estradiol and FSH stimulation, granulosa cells differentiate into mural granulosa cells (MGCs), which line the follicular wall and the fluid‐filled antrum, perform endocrine functions and sustain follicular development, and cumulus cells (CCs),^[^ ^32^ ^]^ cuboidal cells in layers that surround the oocyte and maintain functional coupling with it through gap junctions. These cells support oocyte growth by providing essential nutrients, expand in response to FSH, and produce hyaluronic acid upon ovulation.^[^ ^33^ ^]^ In return, the oocyte secretes specific factors, such as growth and differentiation factor‐9 (GDF‐9) and BMP‐15 (Figure 2), which further promote the proliferation and differentiation of granulosa cells.^[^ ^34^, ^35^ ^]^

During the follicular phase of the ovulatory cycle, FSH induces the formation of the follicular wave, with an initial growth response by several follicles. FSH levels then decline leading to the selection of the dominant follicle, which continues to grow independently from this hormone and then becomes dependent from LH.^[^ ^36^ ^]^ Anti‐Müllerian hormone (AMH) also plays a crucial role in the selection of the dominant follicle. Similarly to FSH, AMH levels decrease as the follicle grows, becoming almost undetectable in the dominant follicle.^[^ ^36^ ^]^

During the preovulatory phase, the LH surge induces the final differentiation of granulosa cells into luteal cells and the shift from estradiol to progesterone production.^[^ ^37^ ^]^ This event leads to a reduction in gap junction coupling, causing the dissociation of mural granulosa cells and the expansion of the cumulus‐oocyte complex. With ovulation, the oocyte resumes meiosis and progresses from prophase I to metaphase II.^[^ ^36^ ^]^ Progesterone blocks the cyclic hormonal regulation of ovarian function in favor of developing and maintaining a pregnancy, stimulating the production of a thick cervical mucus, which inhibits sperm cell movement through the uterus.

Aromatase expression in granulosa cells is tightly regulated by various hormonal and growth factors. During folliculogenesis, aromatase activity in granulosa cells gradually increases, peaking in healthy antral follicles. This activity is strictly dependent on normal gonadotropin levels and can be enhanced by cAMP, which represents the main intracellular messenger of FSH, and growth factors that include TGF‐β and IGF1 (Figure 2).^[^ ^38^, ^39^ ^]^ Other factors, such as prolactin, glucocorticoids, epidermal growth factor (EGF), fibroblast growth factor (FGF), and platelet‐derived growth factor (PDGF), reduce aromatase activity.^[^ ^40^, ^41^ ^]^

## 3 PCOS Pathogenesis

**Figure 3:**
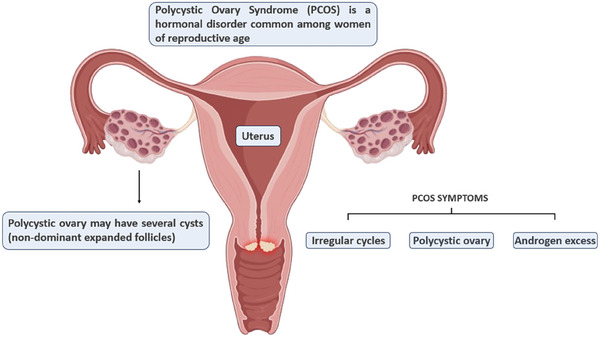
Main symptoms of PCOS. As reported by the Androgen Excess and PCOS Society, the presence of ovarian cysts is not essential for PCOS diagnosis.^[^ ^7^ ^]^ (Composed in BioRender.com).

PCOS is associated with infertility and is characterized by the presence of ovaries with functional abnormalities that include chronic inflammation, oxidative stress, and inhibition of aromatase activity, leading to hyperandrogenism (**Figure** **3**). This condition alters follicle physiology, affecting follicular cells, and results in a greater density of small pre‐antral and antral follicles and a higher percentage of follicles that grow prematurely. As demonstrated in PCOS mouse models, ovarian follicles have a significantly enlarged theca cell layer while the granulosa cell layer is reduced, which is consistent with the characteristics of a hyperandrogenic phenotype.^[^ ^42^ ^]^

Various studies have suggested that dysfunctions in theca cell‐granulosa cell interactions and granulosa cell‐oocyte interactions may contribute to the abnormal folliculogenesis observed in PCOS.^[^ ^43^ ^]^ While PCOS is primarily associated to ovarian dysfunction, central neuroendocrine systems play a crucial role in its pathophysiology. Women with PCOS exhibit elevated LH production, characterized by persistently high LH pulse frequency, increased LH pulse amplitude, and an exaggerated response to exogenous GnRH, and is further accompanied by a relative deficiency in FSH. These abnormalities in gonadotropin secretion are key contributors to the ovarian hyperandrogenemia and ovulatory dysfunction seen in PCOS.^[^ ^44^ ^]^ Insulin also plays a role in the ovary, where it regulates follicle cell functions (Figure 2). Insulin resistance is observed in 50–90% of PCOS patients, leading to hyperinsulinemia.^[^ ^45^ ^]^ Hyperinsulinemia intensifies LH pulses, stimulates adrenal CYP17A1 activity, and reduces hepatic production of SHBG.^[^ ^46^ ^]^ These conditions act synergistically on ovarian theca cells, activating CYP17A1 and increasing circulating androgens. The impact of insulin resistance in PCOS extends to various physiological processes both inside and outside the ovary. Improving insulin sensitivity through weight loss or medication has been shown to alleviate the reproductive, hyperandrogenic, and metabolic symptoms of PCOS. Conversely, weight gain worsens insulin resistance, exacerbating the clinical manifestations of PCOS. The effect of insulin resistance and hyperinsulinemia in PCOS is explained by examining the underlying molecular pathways. Insulin primarily activates the intracellular phosphatidylinositol 3‐kinase (PI3‐kinase) and mitogen‐activated protein kinase (MAP kinase) pathways.^[^ ^47^ ^]^ Moreover, insulin resistance results in an alteration of the PI3‐kinase pathway, leading to compromised metabolic function. This contributes to the observed cellular responses in PCOS, including metabolic dysfunction, increased steroidogenesis, hyperandrogenism, and ultimately reproductive disorders.^[^ ^48^, ^49^ ^]^

Some studies have suggested that autophagy also plays a significant role in the pathogenesis of PCOS. In the ovary, autophagy is important as it regulates events from the formation of the oocyte to its fertilization. Consequently, defective autophagy of follicle cells has been observed in PCOS patients.^[^ ^50^ ^]^

## 4 PCOS Models

The complexity of PCOS phenotypes and the challenges of studying its pathology in humans have driven researchers to develop in vivo animal models and in vitro human ovarian cell models for preclinical research. These models enable the molecular investigation of key mechanisms underlying PCOS, including disruptions in the hypothalamic‐pituitary‐ovarian axis, excessive androgen production by theca cells, and impaired hormonal feedbacks.

### 4.1 Animal Models

Animal species used to develop PCOS models include primates, where PCOS appears to occur naturally, sheep, rodents (i.e., mice and rats), in which the condition does not occur naturally, and non‐mammalian species such as zebrafish.

PCOS models are generally developed by genetic and/or hormonal approaches. Stimulation with androgens such as testosterone, DHEA, and dihydrotestosterone (DHT), to emulate the condition of hyperandrogenism typical of PCOS, is the basic concept to produce in vivo models. For example, models of pre‐/post‐natal testosterone‐induced PCOS are obtained in *rhesus* monkeys. While both treatments mimic ovarian disfunctions, hyperandrogenism, and oligo‐anovulation, the prenatal testosterone exposure also produces the metabolic imbalances typical of PCOS.^[^ ^51^ ^]^ In sheep, prenatal exposure to androgens produces traits characteristic of PCOS with elevated frequency of LH pulses, hyperandrogenism and oligo‐anovulation.^[^ ^52^ ^]^ In zebrafish, exposure of young females to testosterone also produces a PCOS model with follicular growth‐arrest, ovary enlargement, reduced ovulation rate, and a decrease in reproduction.^[^ ^53^ ^]^

#### 4.1.1 Murine Models

Indeed, mice and rats are the elective laboratory species to investigate various aspects of human pathology preclinically. While there are inherent differences in reproductive physiology between humans and rodents, numerous murine in vivo models have been developed over time to explore the pathophysiology of PCOS.^[^ ^54^ ^]^ For example, genetic obesity models have been found to emulate certain features of PCOS, with studies in *New Zealand obese* mice^[^ ^55^ ^]^ and *Zucker rats* ^[^ ^56^ ^]^ among others, providing insights into PCOS. These animals exhibit spontaneous obesity, often coupled with hyperinsulinemia, ovarian cysts, and reduced fertility; however, they do not typically show elevated serum testosterone or LH levels.

Among PCOS models produced by deletion or overexpression of endocrine system‐related genes, aromatase knockout (Ar KO) mice develop hemorrhagic ovarian cysts, increased serum LH levels, and significantly elevated testosterone levels. This model features increased body weight, fat accumulation, and disrupted lipid metabolism, including hypercholesterolemia. In these mice, the rise in LH release is due to estrogen depletion, which stimulates androgen synthesis in the theca cells, and in turn causes the observed metabolic symptoms.^[^ ^57^ ^]^

Transgenic mice expressing a chimeric LH beta (*LHβ*) subunit that includes the C‐terminal portion of the beta subunit of chorionic gonadotropin, exhibit high levels of LH, reflecting a condition of infertility and miscarriages.^[^ ^54^ ^]^ Compared to control mice, these mice also show increased levels of testosterone and estradiol, infrequent ovulation, a prolonged luteal phase, ovarian cyst formation, and granulosa cell tumors.^[^ ^58^, ^59^ ^]^ Additionally, these mice routinely display metabolic disorders, including obesity and elevated insulin levels.^[^ ^60^ ^]^

Currently, hormonal approaches represent the most common strategy for achieving PCOS models. Stimulation with testosterone, DHEA, and DHT hinders normal reproduction and induce a condition similar to human PCOS, characterized by altered menstrual cycle, formation of ovarian cysts, and development of insulin resistance.^[^ ^61^ ^]^ Prolonged administration of DHEA in rats and mice increases the number of atretic follicles and reduces that of corpora lutea compared to controls,^[^ ^62^ ^]^ as also observed in our laboratory.^[^ ^63^, ^64^ ^]^ Furthermore, DHEA‐treated mice exhibit altered collagen deposition, increased lipid droplet accumulation, and expression of steroidogenic enzymes.^[^ ^63^, ^64^ ^]^

DHT induces a PCOS model with hormonal dysregulations, ovarian alterations, and metabolic disorders typically observed in humans.^[^ ^65^ ^]^ Prepubertal administration of DHT in rats and mice has allowed the study of the main ovarian and metabolic features of PCOS, hyperandrogenism, and accumulation of advanced glycation end (AGE) products.^[^ ^66^, ^67^, ^68^, ^69^ ^]^

An alternative experimental model in rodents involves exposure to continuous light for several weeks, inspired by observations that women exposed to altered night light cycles often experience PCOS features, including disrupted menstrual cycles, dysmenorrhea, metabolic syndrome, and insulin resistance. In a prior study, our group exposed female mice to continuous light for 10 weeks obtaining alterations in ovarian structure and follicle growth, the appearance of ovarian cysts, and an abnormal increase in the theca cell compartment coupled with a decrease in the granulosa cell compartment.^[^ ^42^ ^]^ A 10 day‐treatment of these mice with daily administrations of myo‐inositol and D‐chiro‐inositol, natural components of membrane phosphoglycans that act as second messengers of insulin,^[^ ^70^, ^71^, ^72^ ^]^ at a dosage of 420 mg kg^−1^ and a molar ratio of 40:1, resulted in a rapid and near complete recovery from PCOS symptoms, including the restoration of normal ovarian histological features and reproductive functions.^[^ ^42^ ^]^

In that study we observed that inositol formulations containing higher doses of D‐chiro‐inositol worsened the altered ovarian structure produced by the continuous light treatment. Consequently, we developed an alternative androgenic PCOS mouse model by daily administering D‐chiro‐inositol at a dosage of 250 mg kg^−1^, equivalent to 1200 mg in humans, for ten days. Mice undergoing this treatment displayed ovarian cysts, increased testosterone levels, and reduced ovarian aromatase expression.^[^ ^73^ ^]^ These studies^[^ ^42^, ^73^ ^]^ allowed us to confirm at the preclinical level the efficacy of myo‐inositol and D‐chiro‐inositol in treating PCOS but highlighted the need for specific combined formulations containing low dosages of D‐chiro‐inositol, underscoring the importance of carefully evaluating treatments of PCOS patients with D‐chiro‐inositol at high doses or for prolonged times.

The hyperandrogenic phenotype produced by high dosages of D‐chiro‐inositol is due to the activity of this molecule as both an inducer of androgen biosynthesis in theca cells.^[^ ^74^ ^]^ and an inhibitor of estrogen biosynthesis in granulosa cells.^[^ ^73^, ^75^ ^]^

In another study performed on a rat model of PCOS induced with letrozole, Mihanfar et al. (2021) demonstrated that quercetin effectively alleviates irregularities in the estrous cycle, abnormalities in the lipid profile, elevated serum levels of steroid, and insulin resistance.^[^ ^76^ ^]^

In sum, animal models replicate several features of PCOS, facilitating the study of its pathogenesis,^[^ ^77^ ^]^ and can be used to identify novel strategies and targets of therapeutic interventions.

### 4.2 Cell Lines For In Vitro Studies

Cell lines are an effective and convenient alternative for studying the biological pathways of PCOS. They provide a reproducible source for experimentation, can be manipulated in various ways to study specific aspects of the disease, and allow for less ambiguous results. Additionally, in vitro models represent a more ethical alternative to in vivo animal models.^[^ ^78^ ^]^

#### 4.2.1 Human Granulosa Cell Lines

##### COV434 Cells

COV434 cells are an immortalized cell line derived from a primary granulosa cell tumor, featuring several properties essential for the normal function of human granulosa cells.^[^ ^79^ ^]^ They are utilized in ovarian cancer research, for studying follicular steroidogenic activities and cell‐to‐cell interactions.^[^ ^78^ ^]^ COV434 cells exhibit characteristics typical of granulosa cells, such as growth in small follicular aggregates, and the formation of intercellular junctions when co‐cultured with cumulus cells in the presence of an oocyte.^[^ ^79^ ^]^ They produce 17β‐estradiol in the presence of androstenedione, and upon FSH stimulation express apoptotic markers such as Bak, Bad, and Bax, which can be informative in the in vitro study of follicular atresia mechanisms.^[^ ^79^ ^]^ Despite originating from a metastatic tumor, COV434 cells retain morphological and physiological characteristics of normal granulosa cells, making them a valuable model for studying both normal and pathological ovarian conditions, including androgen excess, which is critical in PCOS,^[^ ^78^ ^]^ and oxidative stress.^[^ ^80^ ^]^

##### HGL5 Cells

The HGL5 cell line was established by transforming human luteinized granulosa cells with human papillomavirus strain 16. Although immortalized, these cells retain the typical characteristics of primary granulosa cells,^[^ ^78^ ^]^ and respond to insulin and androgens signaling, which are critical factors in PCOS pathogenesis. HGL5 cells have been used to evaluate the protective effects of the dietary flavonoid isoquercitrin (or isoquercetin) against oxidative stress through inhibition of intracellular ROS generation,^[^ ^81^ ^]^ and to study the effect of the mesenchymal stem cell secretome on steroidogenesis in a PCOS model.^[^ ^82^ ^]^

##### KGN Cells

KGN cells are a human granulosa cell tumor line that retains crucial physiological characteristics of granulosa cells. These cells produce estrogen, progesterone, and other hormones essential for the menstrual cycle, express functional FSH receptors, and synthesize estradiol in response to FSH stimulation. These features make KGN cells a valuable model for studying steroidogenesis in PCOS.^[^ ^83^ ^]^ They are also used to study gene expression changes associated with PCOS, providing valuable insights into various signaling pathways and molecular mechanisms, including the roles of insulin resistance, hyperandrogenism, and inflammatory cytokines within the disorder.^[^ ^78^ ^]^ In a recent experimental study, an in vitro model of PCOS using KGN cells was produced by inducing oxidative stress to explore the beneficial effects of the polyphenol resveratrol. The cells were stimulated with bacterial lipopolysaccharides (LPS) to induce inflammation and oxidative stress, thereby mimicking PCOS conditions. Treatment with resveratrol significantly reduced both inflammation and oxidative stress in these cells, suggesting a potential therapeutic role for resveratrol in managing PCOS‐related symptoms.^[^ ^84^ ^]^

In sum, cellular models provide a reproducible and stable platform to isolate abnormal biochemical mechanisms of PCOS and appear also useful to identify novel therapeutic strategies.^[^ ^78^ ^]^

#### 4.2.2 Human Theca Cell Lines

Theca cells are particularly valuable for studying abnormal mechanisms related to insulin resistance and/or LH imbalance that can lead to abnormal steroidogenesis and hyperandrogenism in PCOS. Although primary cultures of theca cells from women with PCOS have been used to understand several regulatory aspects of steroidogenesis and the mechanisms leading to hyperandrogenism,^[^ ^85^ ^]^ cell lines or tumor‐derived lines are not well‐described in the literature.^[^ ^78^ ^]^

Ovarian tumor cells expressing androgens (human ovarian theca‐like tumor cells) have been tentatively used as a theca cell line to study the molecular mechanisms regulating human ovarian cell steroidogenesis and the expression of steroid‐metabolizing enzymes.^[^ ^86^ ^]^ However, studies using these cells have not been replicated.

#### 4.2.3 Other Steroidogenic Cell Lines

##### H295R Cells

The H295R cell line, also called NCI‐H295R, is an enhanced version of the H295 cell line, derived from a stage II secretory adrenal cortex carcinoma. This cell line synthesizes various classes of steroid hormones, in particular glucocorticoids, mineralocorticoids, and androgens, and responds to pituitary adrenocorticotropic hormone (ACTH), angiotensin II, and potassium ions. It is widely used for studying human steroidogenesis, specifically for the expressional regulation of several steroidogenic enzyme genes.^[^ ^87^, ^88^ ^]^

Since genes expressed in H295R cells do not overlap those expressed in theca or granulosa cells, they cannot be used to emulate cellular functions and interactions occurring in the ovary. Thus, they may not be directly informative for the hormonal changes in PCOS. However, they can be an effective in vitro model for studying molecular androgenic pathways involved in PCOS, complementing data obtained from other models.

## 5 Conclusions, Advantages, Limitations and Future Perspectives

PCOS is a complex endocrine condition, and its mechanisms remain unclear despite extensive research on human patients, animal models, and in vitro human cellular models, warranting further experimental efforts. Studies so far conducted on animal models produced by different approaches allow to discriminate among different phenotypes of PCOS patients depending on organic/environmental factors, such as hormonal imbalances and life habits, and help elucidate cellular, biochemical, and histological alterations produced in the various conditions.

As for novel information on the management of PCOS, these studies have provided insights into the search/use of molecules with effective therapeutic actions such as, for example, quercetin^[^ ^76^ ^]^ or inositols.^[^ ^42^ ^]^ Studies on animal models may also be useful to identify molecules that have a detrimental effect on ovarian functions, such as D‐chiro‐inositol, which was found to reduce ovarian aromatase expression and to be responsible of a PCOS phenotype when provided at high doses.^[^ ^73^ ^]^

The use of cellular model systems allows researchers to avoid the limitation of patient availability and the ethical concerns of animal studies. Moreover, biological variables and treatments can be more precisely controlled in cell cultures than in whole organisms, improving result reproducibility and simplifying the study of cellular and molecular mechanisms. Unlike animal models, human cellular models may provide results that do not need extrapolation to our species. Also considering the advancements in tissue quality and homogeneity, they are a valuable alternative for conducting preclinical studies in human pathology, particularly for PCOS.

Granulosa cell lines constitute valuable models for studying biological functions and steroidogenesis in these cells. The use of these cell lines aids investigation into the effects that various pharmacological agents and natural compounds have on their normal and altered pathways and viability, as shown for isoquercitrin and resveratrol,^[^ ^81^, ^84^ ^]^ thus further leading to the development of potential therapeutic strategies for PCOS.

However, both models have limitations. Animal models often fail to capture clinical manifestations such as menstrual irregularities and metabolic syndrome, complicating the translation of findings to human contexts. Additionally, differences in hormonal responses, reproductive cycles, and metabolic conditions between rodents and humans somehow limit the generalizability of results. Cellular models, while effective for examining molecular aspects of PCOS, cannot replicate the full complexity of ovarian physiology. Additionally, no cell line can exhibit the complete gene expression profile of normal follicle cells, and thus cannot fully replicate the complexity of PCOS.^[^ ^78^ ^]^

New perspectives would require the derivation and development of additional theca cell lines, specifically aimed at obtaining theca‐granulosa cell co‐culture systems. These in vitro systems would better reproduce the ovarian microenvironment, providing more specific information on normal and abnormal paracrine regulation of cell functions, and aid the identification of novel therapeutic targets and strategies for managing PCOS.^[^ ^89^ ^]^

This goal would particularly benefit from integrating genomics, transcriptomics, proteomics data, and analyzing potential epigenetic modifications which may mimic PCOS conditions.

One promising area for new therapies is the potential of regenerative medicine to enhance ovarian health. Stem cells have demonstrated the ability to restore ovarian functions, likely due to the secretion of paracrine factors promoting antioxidant, anti‐inflammatory, and pro‐angiogenic effects.^[^ ^82^, ^90^ ^]^ In our laboratory, we have established in vitro PCOS models using KGN cells exposed to high doses of D‐chiro‐inositol^[^ ^73^ ^]^ or LPS^[^ ^84^ ^]^ and are currently evaluating the beneficial effects of the mesenchymal stem cell secretome.

Finally, since granulosa cells express markers specific to mesenchymal cells, such as CD105, CD90, and CD44, and can differentiate into various cell types like osteoblasts, neurons, and chondrocytes, further research on ovarian cells in culture as a potential source of stem cells for translational medicine represents a promising avenue of research.^[^ ^91^ ^]^

## Conflict of Interest

The authors declare no conflict of interest.

## Author Contributions

A.B., C.G., G.D.E., S.H.M., V.U., and C.T. performed conceptualization; A.B., C.G. wrote the original draft; A.B., C.G., G.D.E. and C.T. and edited. C.G. figure composition.
